# Right ventricular failure due to chronic pressure load: What have we learned in animal models since the NIH working group statement?

**DOI:** 10.1007/s10741-015-9479-6

**Published:** 2015-03-13

**Authors:** Marinus A. J. Borgdorff, Michael G. Dickinson, Rolf M. F. Berger, Beatrijs Bartelds

**Affiliations:** 1 Department of Pediatrics, Center for Congenital Heart Diseases, University Medical Center Groningen, University of Groningen, Groningen, The Netherlands; 2 Department of Cardiology, University Medical Center Groningen, University of Groningen, Groningen, The Netherlands; 3 Department of Pediatric Cardiology, Center for Congenital Heart Diseases, Beatrix Children’s Hospital, University Medical Center Groningen, Antonius Deusinglaan 1, 9713 AV Groningen, The Netherlands

**Keywords:** Pressure overload, Congenital heart diseases, Pulmonary hypertension, Pulmonary artery banding, MRI, Pressure–volume analysis

## Abstract

Right ventricular (RV) failure determines outcome in patients with pulmonary hypertension, congenital heart diseases and in left ventricular failure. In 2006, the Working Group on Cellular and Molecular Mechanisms of Right Heart Failure of the NIH advocated the development of preclinical models to study the pathophysiology and pathobiology of RV failure. In this review, we summarize the progress of research into the pathobiology of RV failure and potential therapeutic interventions. The picture emerging from this research is that RV adaptation to increased afterload is characterized by increased contractility, dilatation and hypertrophy. Clinical RV failure is associated with progressive diastolic deterioration and disturbed ventricular–arterial coupling in the presence of increased contractility. The pathobiology of the failing RV shows similarities with that of the LV and is marked by lack of adequate increase in capillary density leading to a hypoxic environment and oxidative stress and a metabolic switch from fatty acids to glucose utilization. However, RV failure also has characteristic features. So far, therapies aiming to specifically improve RV function have had limited success. The use of beta blockers and sildenafil may hold promise, but new therapies have to be developed. The use of recently developed animal models will aid in further understanding of the pathobiology of RV failure and development of new therapeutic strategies.

## Introduction

Right ventricular (RV) failure is a major determinant of outcome in patients with pulmonary hypertension (PH), corrected congenital heart diseases (CHD) and in left ventricular failure due to ischemic heart disease [1–3]. Improved therapies for these diseases have led to a quickly expanding population of children and young adults at risk of mortality due to RV failure. For example, currently over four million adults in Europe and the USA suffer from late effects after treatment for CHD. Despite the pivotal involvement of the RV in both common and rare cardiovascular diseases, the mechanisms of RV failure have historically gotten little attention. Already in 1988, Reeves pleaded that *“One must inquire how increasing pulmonary vascular resistance results in impaired RV function”* (Reeves, cited in [4]). However, almost 20 years later in 2006, a Working Group on Cellular and Molecular Mechanisms of Right Heart Failure of the National Heart, Lung and Blood Institute concluded that there was *“*a *paucity of basic knowledge at all levels about the RV’s normal and pathological function”* [5]. This observation led to a call to the research community to develop accurate preclinical models, to study the pathophysiology and pathobiology of RV failure and to develop new therapeutic strategies [5]. In this review, we summarize the research in animal models since then into the pathophysiology and pathobiology of RV failure and possible therapeutic interventions.

## Modeling and evaluating a unique ventricle

### The right ventricle is not a mirror image left ventricle

The RV has specific characteristics that affect the response to abnormal loading conditions, as extensively reviewed previously [6]. In short, firstly, the RV is derived from a distinct set of precursor cells (as compared to the LV), the so-called secondary heart field [7]. It is yet unknown whether this different embryological origin affects the response to abnormal loading conditions [8]. Secondly, the RV is a crescent-shaped structure wrapped around the LV that has a unique contraction pattern, which complicates functional and volumetric analysis. Thirdly, the normal RV is unloaded after birth. During fetal life, the RV and LV work in parallel to support the systemic and pulmonary circulation, but after birth, these circulations are serially connected and the pulmonary vascular resistance, which determines the afterload of the RV, progressively decreases. In patients with CHD, this unloading is often absent [9]. Fourthly, the dominant movement of the RV is longitudinal shortening, pressing the RV-free wall against the septum to create a bellows effect to empty into the low-resistance pulmonary circulation [6]. Finally, in normal adult conditions, the RV has a lower oxygen requirement as compared with the LV and lower coronary flow that mostly occurs during systole [10]. A comprehensive overview of differences between the RV and LV from a clinical and preclinical perspective was published recently [11]. In the current paper, we present a detailed analysis of the experimental literature describing the adaptation of the right ventricle in response to increased afterload.

### Evaluation of RV function and failure

To interpret the findings in preclinical models and to translate these to clinical practice, a definition of RV failure is necessary. Right heart failure is not an entity as such but a continuum of disease severities and clinical symptoms and can be defined in congruence with previous definitions of heart failure by Sugawa and Sunagawa [12]. Heart failure is defined as the inability to meet the requirements of the metabolizing tissues of the body. RV failure is defined accordingly, but the clinical signs and symptoms may differ from those in LV failure [13]. The cardinal clinical characteristics of RV failure are low (effective) cardiac output (evident in exercise intolerance, fatigue, dyspnea and poor peripheral circulation) and fluid retention (evident in peripheral edema, effusion and ascites) [6, 13] (Table 1). It is therefore important, in clinical practice but also in preclinical animal experiments, to include these clinical signs in the assessment of RV function or failure, in addition to functional RV parameters.

**Table 1:** Evaluation of RV disease in animal models

Parameter	Examples
Type of loading	Proximal pressure load (e.g., pulmonary artery banding)
	Peripheral pressure load (e.g., pulmonary hypertension)
	Volume load (e.g., aorto-caval shunt, pulmonary/tricuspid valve regurgitation)
	Combined pressure/volume load (e.g., pulmonary hypertension + aorto-caval shunt
Clinical symptoms	Appearance (decreased grooming or inactivity)
	Bodyweight changes (cachexia or fluid retention)
	Cyanosis or decreased peripheral circulation
	Dyspnea/tachypnea (labored breathing)
	Effusions (palpable ascites)
Exercise	Voluntary/spontaneous activity
	Forced exercise testing
Effusion at autopsy	Pleural effusion
	Ascites
	Liver wet/dry weight ratio
Survival	Mortality
	Human endpoints reached

In clinical practice, *exercise capacity* is used as an important guide to grade the severity of heart failure and as a prognostic indicator. Exercise capacity in patients can be determined by maximal cardiopulmonary exercise testing or by voluntary exercise performance, evaluated with a 6-min walk distance. Similarly, in animal models of RV disease, forced exercise capacity can be evaluated by a treadmill test [14, 15] and voluntary exercise capacity by spontaneous activity in a running wheel [8, 16, 17].

*Mortality* is the ultimate clinical sign of RV failure, and survival analysis may be included in studies. However, in animal models, other factors rather than RV failure that might impede survival (such as pulmonary disease and/or LV dysfunction in the monocrotaline model) should be excluded or accounted for [18]. Once RV disease has been characterized using clinical symptoms, exercise and/or mortality (Table 1), the disease state can be coupled to hemodynamic and cellular adaptation (Table 2).

**Table 2:** Overview of hemodynamic changes in models of RV pressure load

Species	Model	Signs and symptoms	Survival	Exercise	RVP	EDP	Ees	Ees/Ea	Eed	CI or CO	EDV	Ref	Remark
*Peripheral pressure load*													
Rat	mct30	None	No mortality	n/a	27	56	21	=	−16	9	13 S	[36]	
Rat	mct80	↓BW, inactivity	No mortality	n/a	67 S	38	15	↓	−9	−26	89 S	[36]	
Rat	mct80	Yes (see R1)	No mortality	V ↓	96 S	199 S	188 S	n/a	4	−8	30	[17]	R1, R2
Rat	mct60	↓BW, resp distress	↑ mortality	n/a	166 S	200 S	400 S	↓	700 S	−60 S	n/a	[34]	
Rat	mct60	n/a	n/a	n/a	325 S	n/a	766 S	=	500 S	−64 S	n/a	[102]	
Rat	mct40	None	No mortality	F ↓	120 S	400 S	n/a	n/a	n/a	−45 S	n/a	[56]	
Rat	mct60	Yes (see R3)	n/a	F ↓	180 S	650 S	n/a	n/a	n/a	−10	n/a	[56]	R3
Rat	mct60	n/a	n/a	F ↓	140 S	333 S	n/a	n/a	n/a	−30 S	19	[32]	
Rat	mct60	n/a	n/a	n/a	160 S	200 S	n/a	n/a	n/a	−25 S	n/a	[27]	
Rat	mct60	n/a	n/a	n/a	110 S	n/a	n/a	n/a	n/a	−60 S	n/a	[69]	
Rat	mct40	n/a	n/a	n/a	110 S	n/a	n/a	n/a	n/a	−29 S	n/a	[103]	
Rat	mct40	n/a	n/a	n/a	121 S	n/a	n/a	n/a	n/a	−39 S	n/a	[99]	R4
Rat	SuHx	n/a	n/a	F ↓	200 S	n/a	n/a	n/a	n/a	−63 S	n/a	[32]	
Rat	SuHx	n/a	↑ mortality	F ↓	222 S	n/a	n/a	n/a	n/a	−42 S	n/a	[88]	
Rat	SuHx	n/a	No mortality	n/a	208 S	n/a	n/a	n/a	n/a	−42 S	n/a	[28]	R5
Rat	SuHx	n/a	n/a	n/a	283 S	n/a	n/a	n/a	n/a	n/a	n/a	[66]	
Rat	FHR	n/a	n/a	F ↓	36	n/a	n/a	n/a	n/a	−42 S	n/a	[104]	
Rat	mct80	↓ BW	No mortality	n/a	n/a	n/a	n/a	n/a	n/a	−50 S	25 S	[51]	
Rat	mct60	n/a	n/a	n/a	n/a	n/a	n/a	n/a	n/a	−83 S	n/a	[105]	
Rat	mct60	n/a	n/a	n/a	126 S	n/a	n/a	n/a	n/a	n/a	n/a	[106]	
Rat	mct60	n/a	n/a	n/a	130 S	n/a	n/a	n/a	n/a	n/a	n/a	[107]	
Rat	mct60	Yes (see R6)	↑ mortality	n/a	133 S	n/a	n/a	n/a	n/a	n/a	n/a	[108]	R6
Rat	mct60	Yes (see R7)	↑ mortality	n/a	133 S	n/a	n/a	n/a	n/a	n/a	n/a	[109]	R7
Rat	mct60	n/a	↑ mortality	n/a	133 S	n/a	n/a	n/a	n/a	n/a	n/a	[110]	
Rat	mct80	Yes (see R8)	No mortality	n/a	n/a	n/a	n/a	n/a	n/a	n/a	n/a	[37]	R8
Rat	mct30	none	No mortality	n/a	n/a	n/a	n/a	n/a	n/a	n/a	n/a	[65]	
Rat	mct80	Yes (see R9)	No mortality	n/a	n/a	n/a	n/a	n/a	n/a	n/a	n/a	[65]	R9
Rat	mct60	n/a	n/a	n/a	n/a	n/a	n/a	n/a	n/a	n/a	n/a	[94]	
Rat	mct60	n/a	n/a	n/a	n/a	n/a	n/a	n/a	n/a	n/a	n/a	[98]	
Rat	mct60	n/a	n/a	n/a	n/a	n/a	n/a	n/a	n/a	n/a	n/a	[83]	
Pigs	AVS	n/a	No mortality	n/a	29 S	n/a	−13	↓	n/a	−44 S	n/a	[81]	R10
Pigs	AVS	n/a	No mortality	n/a	84 S	n/a	74 S	=	n/a	3	n/a	[39]	R11
*Proximal pressure load*													
Lamb	pab > 8	None	No mortality	n/a	433 S	75	281 S	n/a	62 S	−37 S	−13	[42]	R12
Rabbit	pab5	None	No mortality	n/a	271 S	30 S	185 S	n/a	62	n/a	n/a	[58]	
Dog	pab13	n/a	No mortality	n/a	105 S	n/a	243 S	n/a	116 S	0	n/a	[41]	
Rat	pab4	Mild symptoms	No mortality	V ↓	169 S	500 S	162 S	n/a	125 S	−15 S	60 S	[16]	
Rat	pab8	ABCDE (see R13)	↑ mortality	n/a	204 S	300 S	338 S	↓	1053 S	−50 S	n/a	[43]	R13
Rat	pab6	None	n/a	n/a	117 S	40	100 S	n/a	n/a	−5	−18	[26]	
Rat	pab12	None	n/a	n/a	97 S	50	9	n/a	n/a	−25 S	n/a	[111]	R14
Rat	pab20	None	n/a	n/a	113 S	17	−9	n/a	n/a	−12	n/a	[111]	R15
Rat	pab3	n/a	n/a	n/a	166 S	200 S	n/a	n/a	n/a	−26 S	n/a	[27]	
Mouse	pab4	None	No mortality	V ↓	300 S	n/a	n/a	n/a	n/a	0	20 S	[8]	
Rat	pab4	n/a	n/a	F ↓	220 S	n/a	n/a	n/a	n/a	−53 S	n/a	[32]	
Rat	pab7	n/a	n/a	n/a	152 S	n/a	n/a	n/a	n/a	−37 S	n/a	[69]	
Rat	pab6	Yes (see R16)	No mortality	n/a	200 S	n/a	n/a	n/a	n/a	0	n/a	[86]	R16
Rat	pab6	n/a	n/a	n/a	217 S	n/a	n/a	n/a	n/a	n/a	n/a	[66]	
Rat	pab22	n/a	No mortality	n/a	n/a	n/a	n/a	n/a	n/a	0	n/a	[28]	
Mouse	pab3	n/a	n/a	n/a	n/a	n/a	n/a	n/a	n/a	0	75 S	[38]	
Rat	pab4	n/a	n/a	F ↓	n/a	n/a	n/a	n/a	n/a	−42 S	n/a	[15]	
Rat	pab6	n/a	No mortality	n/a	n/a	n/a	n/a	n/a	n/a	0	n/a	[28]	
Rat	pab8	n/a	n/a	F ↓	n/a	n/a	n/a	n/a	n/a	−45 S	n/a	[15]	
Rat	pab lowcu	n/a	n/a	n/a	n/a	n/a	n/a	n/a	n/a	n/a	n/a	[28]	
Rat	pab3	n/a	n/a	n/a	n/a	n/a	n/a	n/a	n/a	n/a	n/a	[50]	
Rat	pab6	n/a	n/a	n/a	n/a	n/a	n/a	n/a	n/a	n/a	n/a	[50]	
Mouse	pab6	Yes (see R17)	↑ mortality	n/a	n/a	n/a	n/a	n/a	n/a	n/a	n/a	[29]	R17
Rat	pab9	n/a	n/a	n/a	n/a	n/a	n/a	n/a	n/a	n/a	n/a	[50]	

Numbers are percentages increase/decrease versus controls. For some studies and parameters, these are approximations depending on how precise data were reported. S indicates a significant change versus controls. Number behind ‘MCT’ indicates the dosage of monocrotaline in mg/kg. Number behind ‘PAB’ indicates number of weeks after which measurements were performed

*MCT* monocrotaline, *PAB* pulmonary artery banding, *FHR* fawn-hooded rat, *SuHx* Sugen–Hypoxia, *AVS* arterio-venous shunt, *BW* bodyweight, *RVP* right ventricular peak or systolic pressure, *EDP* end-diastolic pressure, *Ees* end-systolic elastance, *Ea* arterial elastance, *Eed* end-diastolic elastance, *CI* cardiac index, *CO* cardiac output, *EDV* end-diastolic volume

*for exercise V indicates *voluntary* exercise testing, F indicates *forced* exercise testing; ↓ = decreased; ↑ = increased; == unchanged

Remarks: **R1** Some weight loss, inactivity and dyspnea. **R2** V↓ was trend (*p* = 0.08). **R3** > 5 % loss of body mass a day, lethargy, cyanosis, respiratory distress. **R4** CO estimated on ventricular diameters. **R5** Pericardial fluid on echo; mortality steeply increased after 6 weeks. **R6/R7** BW loss > 10 % for 2 days and arterial oxygen saturation <80 %. **R8** ‘signs of heart failure including pleural effusion and ascites’. **R9** Weight loss, pleural effusion, ascites. **R10/R11** Hemodynamic measurements after shunt clamping. **R12** Duration of PAB varied, but exceeded 8 weeks. Eed is stiffness constant. **R13** All ABCDE categories (see Table 1). **R14/R15** CO is very low in these studies. **R16** Failure symptoms are not defined. **R17** Failure symptoms were: edema, bodyweight changes (both ↑ and ↓). EDP was 300 S after 10 days in the tightest PAB group

### Modeling RV abnormal loading conditions

To study the mechanisms of RV failure, animal models that mimic specific diseases have been developed. The diseases contributing to the spectrum of patients with RV failure can be divided into three main groups: (1) patients with PH, (2) (corrected) CHD and (3) RV failure secondary to left ventricular failure. Although distinct in etiology, these diseases share in common the abnormal loading conditions imposed on the RV, i.e., increased afterload, increased preload or a combination of both [19]. Increased afterload can be peripherally located, as in PH or fixed proximally as in pulmonary stenosis. Either way, the coupling between the RV and the pulmonary arteries is disturbed (Table 2). To represent these chronic abnormal loading conditions models of PH, pulmonary artery banding and/or pre-tricuspid systemic-to-pulmonary shunts have been developed. Knowledge on the RV response to an increase in preload is scarce [16, 20, 21] and will be discussed in a separate paper. In this review, we will focus on the response to increased afterload.

### Models of increased afterload

Historically, researchers used the monocrotaline model (MCT) to induce PH (Table 2a). The MCT model has been invaluable for PH research, but it may be questioned whether rats with MCT-induced PH die from progressive PH, from RV failure or from other organ dysfunction [18]. Besides MCT, other models mimicking PH have been developed, e.g., hypoxia (with or without VEGF inhibitor SUGEN) or the induction of increased pulmonary blood flow [22–25]. Although all these models may represent pulmonary vascular disease adequately, the potential direct effects of the triggers used to induce PH on the RV limit their use to study the pathobiology of RVF.

The *pulmonary artery banding model* (PAB) avoids these limitations of the PH models (Table 2). The banding of the pulmonary artery induces no systemic or toxic effects, and the fixed constriction of the banding ensures a constant afterload, also when pulmonary vasodilators are administered. The time course of the phenotype in this model has shown considerable variability, which may stem from differences in strain, growth rate and size of constriction. Mild constriction will lead to a chronically compensated state with increased RV systolic pressure and RV hypertrophy, but no symptoms of RV failure [16, 26, 27]. Rats have been reported to survive up to 22 weeks in such condition, despite (near) systemic RV pressures [28]. However, a tighter PAB has been demonstrated to lead to clinical symptoms of RV failure, i.e., inactivity, decreased cleaning behavior (raised fur), poor peripheral circulation, dyspnea/tachypnea, ascites and pleural/pericardial effusions and, ultimately, mortality in a high percentage of animals [17, 29–31]. High-intensity exercise capacity [32] and voluntary low-intensity exercise [8, 16, 17] are reduced in this model. These data indicate that a well-sized PAB represents a valuable model of chronic pressure load-induced RV failure.

## The physiology of RV adaptation to increased afterload

The RV response to increased afterload shares several features among all in vivo animal models used. The gold standard to assess the hemodynamic properties of the (loaded) RV is pressure–volume analysis, which allows measurement of several parameters that quantify systolic and diastolic function regardless of loading conditions.

**Fig. 1:**
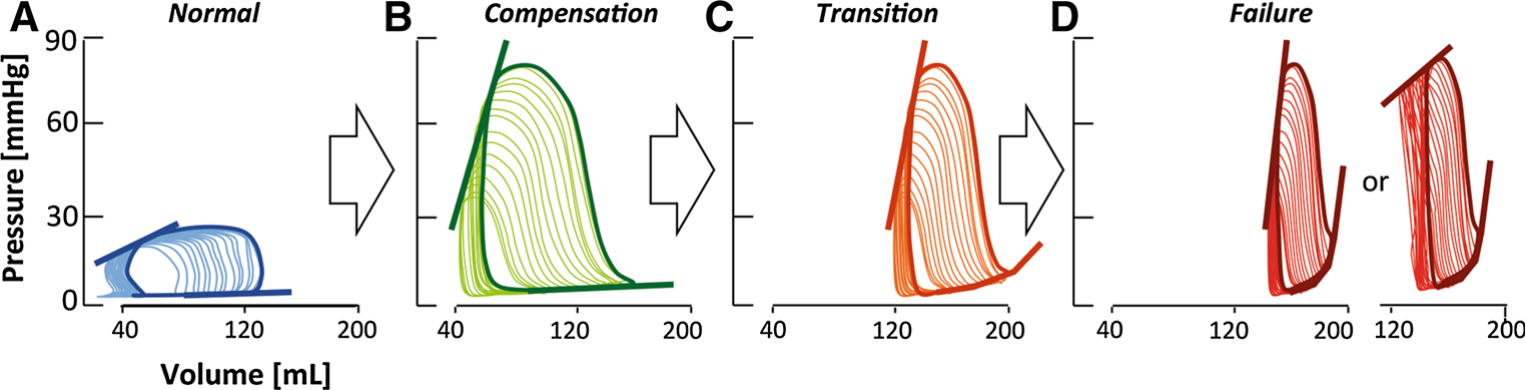
Pathophysiology of the pressure-loaded RV. Conceptual representation of the progression of pathophysiological changes in the pressure-loaded RV. Typical pressure–volume (PV) loops from compensation to failure. Volumetric changes were derived from experimental studies and extrapolated using previously published normal values. *Straight lines* represent the end-systolic elastance (Ees), *dotted lines* represent the end-diastolic elastance (Eed). **a** PV loop in the unloaded RV, showing normal systolic and diastolic function. **b** PV loop in compensated RV, showing increased systolic function (Ees) and RV dilatation (increased end-diastolic volume) but normal diastolic function (Eed). **c** PV loop in transition to failure showing increased systolic function (Ees) and impaired diastolic function (Eed). **d** PV loop in RV failure showing increased or pseudo-normalized systolic function (Ees) and further impaired diastolic function (Eed)

The primary response of the RV to match increased afterload is increased contractility, which is defined as end-systolic elastance (Ees) (Table 2). Contractility increases proportionally with increased arterial elastance (Ea, reflecting afterload) to maintain stroke volume (Fig. 1a, b: typical example of PV loops in response to pressure load). When end-systolic elastance increases less than arterial elastance, the Ees/Ea ratio decreases leading to ventricular–arterial uncoupling, which is regarded a physiological sign of RV failure. Indeed, beneficial pharmacological effects in the pressure-loaded RV are not seldom accompanied by a restoration of Ees/Ea ratio [16, 33, 34].

In chronic pressure load induced by pulmonary artery constriction or experimental PH, increased contractility [35] is accompanied by ventricular dilatation (Fig. 1b; [8, 16, 17, 32, 36–38]). This suggests that also the Frank–Starling mechanism contributes to the RV adaptation to chronic pressure load. Indeed, in most animal models of chronic increased pressure load, ventricular dilatation is associated with decreased Ees/Ea ratio, with the exception of mild monocrotaline-induced PH [36] and 3-month duration of flow-induced PH [39]. Unfortunately, ventricular dilatation leads to increased wall stress [8, 40] and is proposed as a hallmark of the failing ventricle [5], rather than an adaptive response. Improvement of the RV–PA coupling ratio does universally lead to reduction in RV dilatation (Table 3) and wall stress.

**Table 3:** Treatments for RV failure

Group	Agent	Clinically available?	Exercise	Symptoms	Mortality	Contractility	Ees/Ea ratio	Diastolic function	RVH	Fibrosis	Tested in fixed afterload?	Ref
Beta-blockade	Carvedilol	Y	n/a	n/a	Improved	n/a	n/a	n/a	Reduced	Reduced	N	[88]
	Metoprolol	Y	n/a	n/a	Improved	n/a	n/a	n/a	Unchanged	Reduced	N	[88]
	Bisoprolol	Y	n/a	Improved	Improved	Improved	Increased	Improved	Unchanged	Reduced	N	[34]
RAAS inhibition	Losartan	Y	n/a	n/a	n/a	Unchanged	Increased	Improved	Unchanged	n/a	N	[102]
	Telmisartan	Y	n/a	n/a	n/a	n/a	n/a	n/a	Reduced	Reduced	N	[112]
	Losartan/eplerenone	Y	Unchanged	Unchanged	Unchanged	Unchanged	Decreased	Unchanged	Unchanged	Unchanged	Y	[43]
PKG-1-PDE5 pathway	Sildenafil	Y	n/a	n/a	n/a	n/a	n/a	n/a	Unchanged	Unchanged	Y	[50]
	Sildenafil	Y	n/a	n/a	n/a	n/a	n/a	n/a	Unchanged	n/a	Y	[27]
	Sildenafil	Y	Improved	Improved	n/a	Improved	Increased	Unchanged	Unchanged	Increased	Y	[16]
	Sildenafil	Y	Unchanged	Unchanged	n/a	Unchanged	Unchanged	Improved	Unchanged	Reduced	Y	[44]
	Sildenafil	Y	n/a	n/a	n/a	n/a	n/a	n/a	Unchanged	n/a	N	[103]
	Riociguat	N	n/a	n/a	n/a	n/a	n/a	n/a	Reduced	Reduced	N	[95]
	BAY 41-2272	N	n/a	n	n	n/a	n/a	n/a	Unchanged	Unchanged	Y	[113]
Endothelin receptor blockade	Bosentan	Y	n/a	n/a	n/a	n/a	n/a	n/a	Unchanged	n/a	N	[99]
Anti-oxidant	Protandim	N	n/a	n/a	n/a	n/a	n/a	n/a	n/a	Reduced	N	[28]
Anti-oxidant	EUK-134	N	n/a	Unchanged	n/a	n/a	n/a	n/a	Unchanged	Reduced	N	[51]
Rho-kinase inhibitor	Fasudil	N	n/a	n/a	n/a	n/a	n/a	n/a	Reduced	n/a	N	[103]
HDAC inhibitor	Trichostatin A	N	n/a	n/a	n/a	n/a	n/a	n/a	Unchanged	Increased	Y	[86]
Fatty acid oxidation blockade	Trimetazidine	Y	Improved	n/a	n/a	n/a	n/a	n/a	Reduced	n/a	Y	[15]
	Ranolazine	Y	Improved	n/a	n/a	n/a	n/a	n/a	Reduced	n/a	Y	[15]
PDK inhibitor	Dichloroacetate	N	n/a	n/a	n/a	n/a	n/a	n/a	Reduced	n/a	Y	[69]
Estrogen receptor-beta agonist	Genistein	N	n/a	n/a	Improved	n/a	n/a	n/a	Reduced	n/a	N	[106]

Overview of experimental studies reporting on (direct) RV effects of medical treatments in the pressure-loaded RV

*RVH* right ventricular hypertrophy, *HDAC* histone deacetylase, *PDK* pyruvate dehydrogenase kinase, *Ees* end-systolic elastance, *Ea* arterial elastance

Evidence is accumulating that RV failure due to chronic pressure load is characterized by both enhanced systolic function *and* progressive deterioration of diastolic function [16, 31, 34]. Sparse clinical data show that in PH patients, higher right atrial pressure (an indirect measure of RV diastolic function) is associated with worse outcome. This could indicate that diastolic dysfunction contributes to RV failure in patients. From experimental models [41–44] using PAB as afterload (Table 2), it is clear that diastolic dysfunction is an inherent component of increased RV afterload (Fig. 1c, d; [17]). In dogs with a PAB, the diastolic dysfunction of the RV is partly compensated for right atrial adaptation, i.e., right atrial contractility increases, and the right atrium dilates to serve as a reservoir [41]. In a recent study in rats with a PAB separating those with clinical signs of severe RV failure from those without clinical signs but with RV dysfunction, clinical signs of RV failure were associated with a further deterioration of diastolic function despite increased systolic function [31]. These observations are confirmed by studies on isolated myocytes from patients with end-stage RV failure due to PH [45]. Intriguingly, in a recent study in which the RV was subjected to isolated volume load (pulmonary valve regurgitation), diastolic dysfunction was described without changes in Ees [46]. Deterioration of diastolic function might thus play a central role in the transition from compensation to failure (Fig. 1).

Diastolic function has a passive and an active component. The RV (compared to the LV) has been suggested to be particularly vulnerable to disturbed active relaxation, possibly due to insufficient expression of the sodium-calcium exchanger (NCX) [47]. Indeed, in experimental MCT-induced PH, active relaxation is increasingly disturbed with increasing MCT dose [36], while passive stiffness has been reported as normal [17, 36] or increased [48]. Chronic PAB invariably leads to diastolic dysfunction, with disturbances in both active relaxation and passive stiffness [16, 17, 41, 42].

### Studies without pressure–volume analysis

Interpretation of the data in the literature is hampered by the lack of pressure–volume analysis in many studies. The alternatives, CMR-derived RV volumes or echocardiographic parameters for systolic function, only indirectly relate to contractility and are dependent on pre- and/or afterload. Systolic displacement of the lateral tricuspid annulus along the base-apex axis (TAPSE) is decreased in chronic pressure load. This reflects reduced longitudinal movement of the RV, but does not relate to RV pumping function, i.e., shifting volume at a certain pressure [49]. Fractional shortening of the RV outflow tract [29] and change in surface area [50] are also reported, but their significance in the setting of abnormal RV loading is unknown. CMR, the gold standard for RV function in clinical practice, has the advantage of being noninvasive, but without simultaneous pressure measurements, only yields load-dependent variables [8, 51, 52]. RV ejection fraction (EF), another outcome parameter, is generally reduced before overt RV failure ensues, although this parameter is of limited value as it is preload dependent [8, 36]. As described above, PV analysis characterizes the initial RV response as increased contractility and ventricular dilatation, two processes that have opposite effects on EF. Depending on the relative size of changes, EF may thus be increased, decreased or remain unchanged. Recently, Vanderpool et al. [53] suggested RV stroke volume divided by the end-systolic volume as an alternative, less preload dependent parameter.

### Proximal- versus peripheral-type pressure load

Whether the type of pressure load (PAB vs. PH) determines the RV response is a matter of debate as conflicting data have been reported. Echocardiographic data suggest that at similar levels of pressure overload, the RV is less dilated and has superior function in patients with pulmonary stenosis compared with those with PAH. In contrast, a recent experimental comparison between rats with PAB and PH [17] showed that the PAB rats with moderate RV dysfunction (assessed by exercise and clinical signs and symptoms) had more severe RV dilatation in response to afterload than rats subjected to MCT-induced PH.

### Interaction with LV function

The RV and LV functionally interact via shared fibers and the interventricular septum [11]. Interaction of the pressure-loaded RV with LV function has been noted in patients and animal models with PH [54, 55]. In a study in isolated hearts from rats with MCT-PH, pacing improved RV function and reduced interference in the diastolic phase [56]. Also, studies in isolated hearts suggested that the LV contributes to 65 % of the work of the normal RV [57]. In a PAB rabbit model of moderate RV dysfunction, increasing LV afterload via aortic constriction improved systolic RV function [58]. End-diastolic elastance tended to increase, but this effect was not significant.

The increased knowledge on the functional characterization of RV response to increased afterload leads to the question: Can we manipulate this response to support the failing RV? To answer this question, the hemodynamic adaptation has to be coupled to the pathobiology.

## Pathobiology of RV failure

**Fig. 2:**
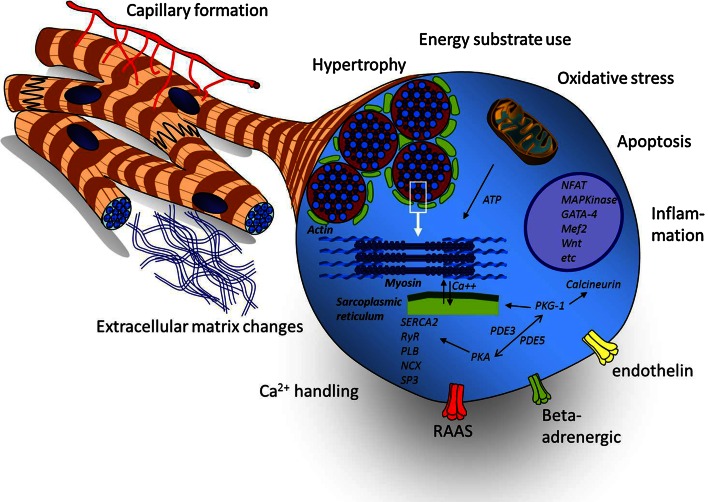
Overview of the pathobiological changes in the abnormally loaded RV. Pathobiological hallmarks of the abnormally loaded RV. A myriad of genetic and epigenetic changes result in tissue damage-related processes (oxidative stress, fibrosis and apoptosis) and activation of (mal)adaptive processes on the tissue (capillary formation, inflammation) or cellular level (hypertrophy, energy substrate use, mitochondrial function and calcium handling). These processes are regulated by a complex network of signaling pathways related to contactile function, cellular growth, energy metabolism and neurohumoral signaling. *ATP* adenosine triphosphate, *NFAT* nuclear factor of activated T cells, *MAPK* mitogen-activated protein kinase, *Mef*2 myocyte enhancer factor-2, *PKG*-1 protein kinase G-1, *PDE*3/5 phosphodiesterase-type 3/5, *PKA* protein kinase A, *SERCA*2 sarcoplasmic reticulum Ca2 + -ATPase, *RyR* ryanodine receptor, *PLB* phospholamban, *NCX* sodium-calcium exchanger, *SP*3 transcriptional repressor SP3, *RAAS* renin angiotensin aldosterone system

In the pressure-loaded RV, a myriad of cellular changes takes place that may initially serve as adaptive remodeling, but are also present in the failing RV (Fig. 2). Rather than caused by a single ‘malignant’ pathway, RV failure is the resultant of many biological changes, both adaptive and maladaptive. The interpretation of these changes is influenced by the state of RV dysfunction assessed by clinical signs and hemodynamic measurements as well as the trigger inducing the afterload (e.g., MCT or PAB). Some of these changes resemble those found in LV remodeling in adaptation to stress [40, 59].

### RV hypertrophy, isoform switch and fibrosis

RV *hypertrophy* is an adaptive response to reduce wall stress and improve contractility. In the LV, hypertrophy is a strong predictor for outcome [59], but in experimental RV failure, this relation is less clear. In all studies in experimental animals, RV hypertrophy is present but this is not related to functional adaptation [16, 17, 26, 28]. In one study, RV hypertrophy was more severe in a PH model than in a PAB model [28], in other studies it was not, possibly due to differences in the degree of loading [14, 17, 26]. This is important to note, as some authors compare different models using the degree of RV hypertrophy to classify the state of adaptation, which may yield contradictory results [15].

Increased afterload of the RV induces a *switch in myosin heavy chain* (MHC) isoform composition from the fast alpha-MHC to the slower but energetically favorable beta-MHC in the RV myocardium [8, 16, 17, 27]. This response is not related to the degree of RV dysfunction. The hypertrophic response is induced by similar pathways as in the LV, e.g., the calcineurin−NFAT pathway [8], which may render the RV a putative target for calcineurin interference or other therapeutic strategies that have been shown to target LV hypertrophy [20].

RV *fibrosis* in response to increased afterload has been reported in some PH models [28, 34, 60] as well as in PAB models [17, 28], although there is a wide variation in the amount of collagen measured across models and the timeframe in which it develops. Given the putative relation between fibrosis and diastolic dysfunction, fibrosis may be a therapeutic target. However, a recent study shows there was no relation between the amount of (interstitial myocardial) fibrosis and the degree of RV dysfunction [17]. One may hypothesize that RV fibrosis is a by-product of the (mal)adaptive RV response to pressure load and has limited pathophysiological significance. Either way, fibrosis can be reduced with several interventions, i.e., beta-blockade [34], ROS scavenger [51], prostacyclin [60], but whether this is a direct effect or secondary to afterload reduction is unclear. Interestingly, an effective strategy to target specifically fibrosis (in LV failure) did not reduce fibrosis nor improve RV function in experimental RV failure [43]. Fibrosis may thus have ventricular-specific characteristics that require further exploration of its pathophysiological significance and therapeutic amenability in RV failure.

### RV capillary formation/RV oxygen supply

Capillary rarefaction can play an important role in the development of RV failure. In the unstressed RV, coronary perfusion is present throughout the cardiac cycle [10], but in patients with PH, the coronary perfusion occurs primarily during diastole [61]. Because RV oxygen consumption also increases; a chronic oxygen demand–supply mismatch may be an underlying mechanism in the transition to failure. Coronary flow has not been measured yet in experimental models, but capillary density was reduced in several PH models [28, 34, 60]. Recently, two studies in various pressure load-induced disease states, i.e., MCT [14] and PAB [31], reported increased capillary density in compensated RV hypertrophy and pseudo-normalized density in decompensated RV hypertrophy. Further support for the significance of insufficient myocardial perfusion comes from the observation that prostacyclin therapy in rats with PH improves both capillary density and survival in the absence of beneficial effects on pulmonary hemodynamics or vascular remodeling [60]. Given its putative importance in the pathophysiology of RV failure, it is questioned which cellular processes are most affected by reduced myocardial perfusion. Candidate pathways are substrate metabolism, mitochondrial function and calcium handling.

### Cardiac metabolism

In the LV, significant shifts in myocardial metabolism have been reported during the transition from adaptation to failure. Fatty acids are the main substrate for the adult heart under normal conditions, but under stress, a switch in substrate use occurs toward glucose and lactate [62]. In addition, glucose metabolism shifts from complete oxidation via the Krebs cycle to glycolysis only, which yields less ATP but also uses less oxygen per ATP molecule. Metabolic shifts are also observed in the pressure-loaded RV. Fatty acid oxidation (FAO) is reduced in PH patients, but only in those with severe hypertrophy [63]. Also, in rats with MCT-induced PH, the expression of CPT-1b (a rate limiting enzyme in the uptake of long-chain fatty acids [64]) was reduced [65, 66]. A reduction in the use of fatty acids seems to be an adaptive mechanism, since preventive inhibition via trimetazidine (which inhibits an essential step in oxidation of FA), increased cardiac output measured by echocardiography in a model of mild RV dysfunction due to a PAB [15].

In addition to the reduction in FAO, patients with PH have an increased uptake of the glucose analog ^18^FDG in the RV [67]. Also, in rats with MCT-induced PH, the expression of glycolysis-related genes is increased [68] as well as the enzymatic rates of glycolysis [69]. Increased expression of glycolysis-related genes has been shown in rats with MCT-PH [69], hypoxic-PH [70], Fawn-Hooded rats [71] and rats with a PAB [15, 69, 72], but none of the rats in these experimental studies had clinical symptoms of RV failure. Similarly, upregulation of pyruvate dehydrogenase kinase (PDK), which uncouples glycolysis from the Krebs cycle, has been shown in both PH and PAB models of adaptive RV remodeling [71]. Inhibition of PDK has been shown to increase RV O_2_ consumption and improve RV function, although a concomitant reduction in RV systolic pressure in the setting of fixed afterload (PAB model) in this study complicated the interpretation of these results [71]. Further evaluation of these effects in models of more severe RV dysfunction is needed since this therapy has also been shown to reverse pulmonary vascular remodeling in experimental PH [73], which could indicate a therapeutic strategy that may benefit both the RV and pulmonary vasculature.

### Mitochondrial function

Mitochondria are the “powerhouses” of the cardiomyocyte, but also regulate many processes involved in the response to stress: formation of oxygen radicals, oxygen sensing, induction of apoptosis and inflammation [74]. The first notion that the RV in PH may be subjected to increased “oxidative stress” came from observations in rats with MCT-induced PH. These rats had increased myocardial activity of complex II and oxygen radicals, and also increased production of radical scavengers [37]. Treatment with a radical scavenger (EUK-134) improved RV systolic function but did not affect diastolic dysfunction [51]; however, in this study, pulmonary vascular resistance was also decreased after EUK-134 treatment, which may have contributed to the beneficial RV effects [75]. In another PH model (SUGEN + hypoxia), treatment with protandim (a plant extract inducing nrf2 expression) increased cardiac output, suggesting that at least in PH rats, increasing defense mechanisms against oxidative stress may be beneficial [28]. These pathways have not been tested in PAB models, i.e., independent from the pulmonary circulation.

### Apoptosis and inflammation

Apoptosis and inflammation, both important mechanisms in the vascular pathology of PH [76–78], have not been explored in detail in the myocardium of models of RV pressure load. However, cardiomyocyte apoptosis and inflammation have been reported in both PH and PAB models [58, 79–81]. Pressure load is linked to apoptosis in multiple ways (mechanic damage, oxidative stress and neurohumoral signaling) and even mildly increased rates of apoptosis can contribute to heart failure [40]. Inflammation, marked by the presence and activation of immune cells and increased activity of inflammatory cytokines also connects pressure load and apoptosis. In addition, in LV pressure, mediators such as TNF-alpha and interleukins interact with neurohumoral signaling, induce fibrosis and affect contractile function and myocardial gene expression [40]. Data on the functional importance of inflammation in the development of RV failure are lacking.

### PKG−PDE5 pathway

The importance of the protein kinase G (PKG) and phosphodiesterase 5 (PDE5) pathway in LV remodeling was shown in mice with transverse aortic constriction [82]. PDE5 catabolizes cGMP, which activates PKG and generally suppresses proliferative pathways. Hence, PDE5 inhibitors (e.g., sildenafil) may enhance the protective effects of PKG. Patients with increased RV afterload showed increased PDE5 expression [83], but only few studies in rat report data on PDE5 expression or PKG activity. PKG activity is not uniformly lowered in all models of experimental RV afterload [16]. Therefore, currently little is known about the importance of this pathway in RV failure. Positive results of intervention studies targeting this pathway (see *Treatment of RV failure* below) underline the importance of more detailed exploration of the PKG−PDE5 pathway in the RV as it may provide new treatment options for RV failure.

### Other pathways

What are the consequences of the unique embryologic origin of the RV for the response to stress [8, 84]? In a rat model of PAB, the expression of dHand, an RV-specific precursor, as well as GATA-4, MEF2 and NKX2.5 were increased [85]. On the other hand, also signaling pathways involved in LV remodeling, such as the calcineurin pathway, have been shown to be activated in a murine model of PAB [8]. However, suppression of calcineurin activation in transgenic mice induced RV dilatation even without pressure load [20]. Similarly, inhibitors of histone deacytelases, which reduce adverse remodeling and improve function in experimental LV pressure load, worsen RV function in rats with a PAB [86]. Such incidental reports suggest that the regulation of adaptation to stress is indeed (partly) chamber specific.

The use of microarrays yields divergent results on genes and pathways possibly involved in the development of RV failure [31, 38, 65, 72, 87]. This may be due to the differences in models used (e.g., MCT vs. PAB), species differences (rat, mouse and rabbit) and different degrees of RV adaptation (compensated vs. decompensated). Additionally, array studies have provided evidence for chamber specificity of gene expression. Table 4 provides a summary of differences in signals between RV and LV in response to increased afterload.

**Table 4:** Differences in signals between RV and LV in response to increased afterload

Model	Mechanism	Difference with LV	Ref
Mouse PAB	Extracellular matrix proteins	↑ expressed in RV	[38]
	Proteases and inhibitors	↑ expressed in RV	[38]
	Developmentally regulated proteins	only expressed in RV	[38]
Rat PAB	PINK1	↓ in RVF, ↑ in LVF	[31, 114]
Mouse PAB vs TAC	miRNA 28,148a,93	↑ in RVF (in non-myocyt fraction)	[87]
Mouse PAB	Wnt signalling	↑↑ in RVF > LVF	[29]
Rat MCT	Mef2c	↑ compensated RV, ↓ RVF, no change LVF	[115]

Overview of studies reporting differences in myocardial signaling between the RV and LV, in response to increased afterload

*PAB* pulmonary artery banding, *TAC* transverse aorta constriction, *RVF* RV failure, *LVF* LV failure

A study in mice, comparing pressure load of the RV via PAB with pressure load of the LV via aortic constriction, showed differences between the two ventricles in expression of genes involved in (1) extracellular matrix proteins, (2) proteases and inhibitors and (3) developmentally regulated proteins [38]. In a similar study, four microRNAs were upregulated in the pressure-loaded RV, but these were all located in the non-myocyte fraction of the RV [80]. Gain/loss of function studies is the next step to determine the functional relevance of these findings.

## Treatment of RV failure

Currently, no RV-specific medical treatment strategies exist. Reports of experimental RV drugs (Table 3) fall into one of three categories: (1) treatment strategies for LV failure (e.g., beta-adrenergic blockade, RAAS inhibition), (2) drugs that target the pulmonary vasculature in PH (e.g., PDE5 inhibitors, endothelin antagonists) and (3) proof-of-concept studies with experimental treatments.

### Beta-adrenergic blockade and RAAS inhibition

The cornerstones of treatment of LV failure are inhibition of the beta-adrenergic receptors and the renin−angiotensin aldosterone system (RAAS). Clinicians are reluctant to prescribe beta blockers to patients with failing RVs in the setting of PH because of their negative inotropic effects, but preclinical studies have shown beneficial effects on RV remodeling. In the hypoxia + SUGEN model of PH, carvedilol, a non-selective beta blocker with also alpha-1-receptor blocking effects, reduced the development of hypertrophy, fibrosis, capillary rarefaction and attenuated reduction in cardiac output and TAPSE [88]. After treatment with Metoprolol, a selective beta blocker, similar effects were observed, although these can be attributed to reduction in the pulmonary vascular remodeling [88]. In the MCT model, bisoprolol (another selective beta blocker) does not prevent hypertrophy or capillary rarefaction, but prevents fibrosis along with delaying the decline in cardiac output and TAPSE [34]. Interestingly, bisoprolol increased phosphorylation of myocardial titin, suggesting a direct RV effect. In conclusion, beta blockers have variable effects in PH models. Unfortunately, no data are published on beta-blockade in the failing RV due to stenosis-type pressure overload, which might elucidate the direct protective effects of beta-blockade on the RV.

Clinical data in PH suggest that the RAAS is activated in at least a subgroup of patients. There are also suggestions that the RAAS system is active in congenital heart disease, and that combinations of RAAS-inhibiting drugs that are used in the standard care for LV failure patients (such as angiotensin receptor blockers + eplerenone) might potentiate the effect and target oxidative stress, fibrosis and improve diastolic dysfunction like in the LV. However, recently no effect of RAAS inhibition was shown in a rat model of PAB [43], in accordance with the results from a study in patients with a systemic RV in CHD [89].

### PDE5 inhibitors

PDE5 inhibition would be an excellent therapeutic approach for RV failure in PH, as it also reduces pulmonary vasculature resistance [90]. However, the first two studies reporting on PDE5 inhibition in the PAB model showed no prevention or reduction in hypertrophy and fibrosis [27, 50]. In these studies, the functional analysis was limited to thermodilution-measured cardiac output and echocardiography. Using pressure–volume analysis, it was shown that preventive sildenafil increases contractility, reduces dilatation and attenuates the decline of spontaneous exercise, but leaves diastolic function unchanged [16], whereas in established RV pressure load, sildenafil treatment predominantly improved diastolic dysfunction, with a small effect on contractility [44]. The differences between the first and the latter studies may be due to differences in the severity of the loading condition, as in the LV it was shown that the effects of PDE5 inhibition are dependent upon the severity of the loading condition [91].

**Fig. 3:**
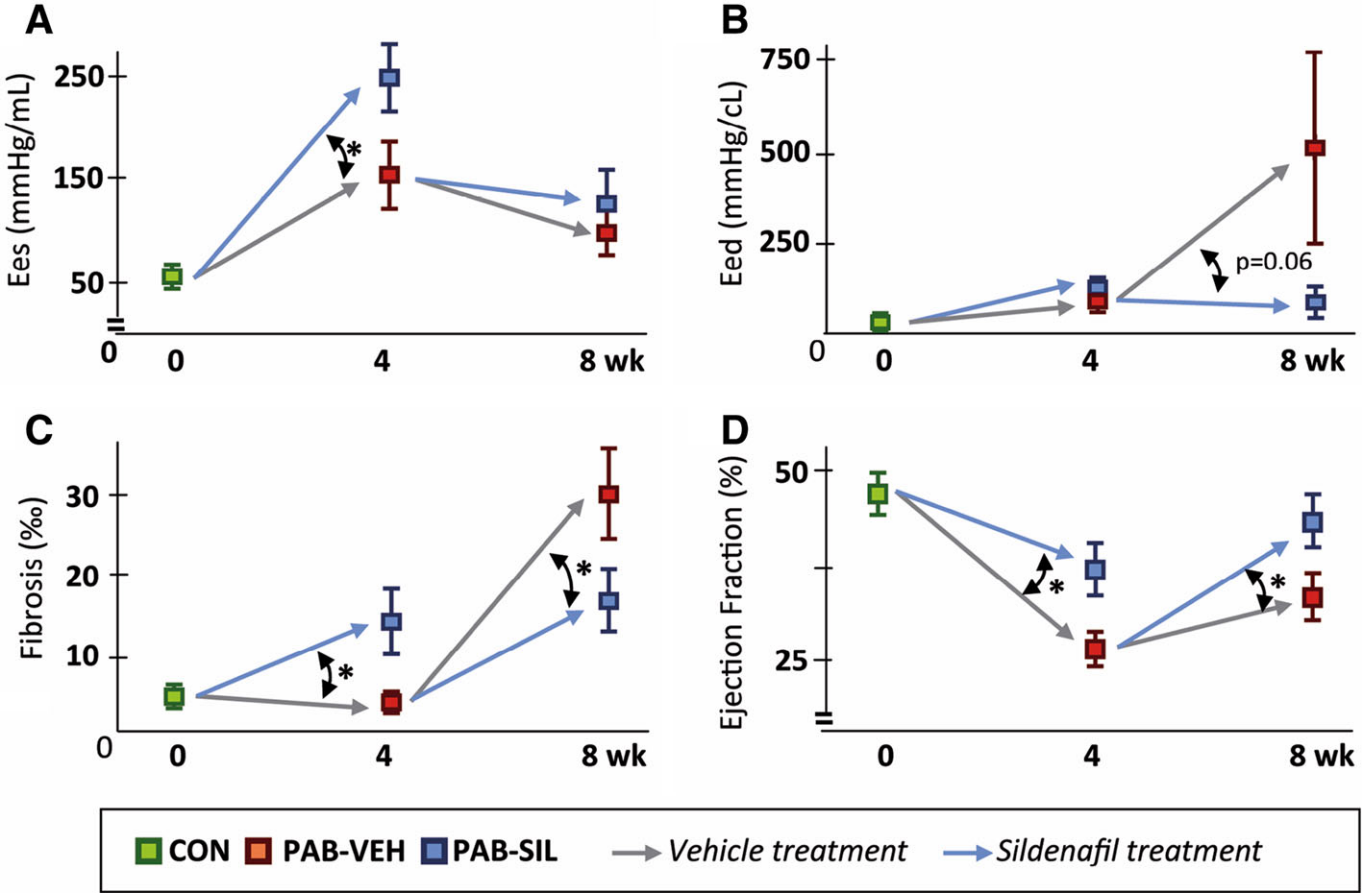
Effects of sildenafil depend on the stage of pressure load-induced RV dysfunction sildenafil effects in the early (0–4 weeks) and late (4–8 weeks) stage of pressure load-induced RV dysfunction are compared. These data illustrate the concept that the effects of pharmacological intervention in the pressure-loaded RV (in these studies with sildenafil) depend on the stage of RV failure when treatment is started. Data of rats with a PAB at week 4 are derived from [16], in which sildenafil treatment was started at the day of surgery (preventive strategy); data of rats with PAB at week 4–8 are derived from [44], in which sildenafil treatment was started 4 weeks after the PAB surgery, when RV dysfunction was already present (therapeutic strategy). **a** End-systolic elastance (Ees). **b** End-diastolic elastance (Eed). To allow comparison of Eed between the studies, the Eed of the initial 2 mmHg end-diastolic pressure drop during occlusion was used here (see methods on characterization of diastolic function). **c** Permillage RV fibrosis per unit surface area. **d** Ejection fraction. Mean ± SEM, * *p* < 0.05. *PAB* pulmonary artery banding, *VEH* vehicle treated, *SIL* sildenafil treated. Figure adapted from [44]; used without permission

The mechanisms by which sildenafil exerts its effects are pleiotropic, incompletely known and not limited to the PDE5−PKG system. Mechanistically, sildenafil inhibits PDE5, increases compartmentalized cGMP and thereby activates PKG-1, which in turn has inhibitory effects on pathways of pathological remodeling [92]. PKG-1 also phosphorylates titin, thereby reducing stiffness [93]. In the pressure-loaded RV, sildenafil treatment as a preventive strategy increased fibrosis [16, 27, 50]. However, when given in a later stage of established pressure load, sildenafil reduced fibrosis and reduced ventricular stiffness [44], supporting the concept that timing and loading severity determines cardiac response (Fig. 3). Sildenafil also stimulates the PDE3−PKA pathway and targets mitochondrial (k)ATP channels, mitochondria and inflammation [94]. Other pharmacological approaches to manipulate the PDE5−PKG-1 axis include stimulation of soluble guanylate cyclase by riociguat, but results so far indicate effects on the pulmonary vasculature, rather than direct beneficial RV effects [95].

### Endothelin receptor antagonists

Endothelin receptor antagonists (ERA) have an anti-hypertrophic and anti-fibrotic effect on the RV in PH [96]. It is unclear whether this is a direct effect on the RV or a consequence of the vasodilatation and anti-proliferative effect on the pulmonary vasculature. Unfortunately, no studies have addressed this issue. Endothelin-1 increases contractility in mouse RV cardiomyocytes via an increase in intracellular Ca^2+^ transients through activation of the Endothelin Receptor-A and the Na^+^–Ca^2+^ exchanger [97]. Upregulation of endothelin-1 in RV myocardium of patients and models of compensated hypertrophy [98] suggests a potential rationale for ERAs. However, isolated heart studies showed that ERAs suppress both contractility and relaxation in the hypertrophic RV [98] which might explain the negative outcomes of preclinical studies on ERAs in RV pressure load without pulmonary vascular remodeling [99]. Intriguingly, the negative inotropic effect of ERAs is absent in LV failure, which may be due to differences in endothelin-1 activation. It may even be possible that ERAs have contrasting therapeutic effects in compensated and failing RVs, but so far, this has not been studied.

Apart from medical intervention, exercise training may be beneficial for the pressure-loaded RV. In patients with a pressure-loaded RV, exercise training induces a modest increase in exercise capacity [100]. In rats with MCT-induced PH; however, exercise training was beneficial in mild PH and deleterious in severe PH [101]. Exercise training has not been tested yet in other models of RV load.

## Conclusion

Since the Working Group Statement, progress has been made in understanding the pathophysiological and pathobiological mechanisms of RV failure due to chronic abnormal loading conditions, specifically increased afterload. The RV adaptation to increased afterload is characterized by increased contractility, dilatation and hypertrophy, whereas clinical RV failure is associated with progressive diastolic deterioration and despite increased contractility, disturbed ventricular−arterial coupling. The pathobiology of the failing RV has several characteristic features. An important factor seems to be the lack of adequate increase in capillary density leading to a hypoxic environment and oxidative stress. Additionally, there is a metabolic switch from FA to glucose utilization in the process of RV adaptation, but the role of this switch in RV failure is yet unclear. So far, therapies aiming to specifically improve RV function have had limited success. The use of beta blockers and sildenafil may hold some promise, but new therapies have to be developed. Finally, a lot of insight has been gained with regard to the specific values and limitations of the different models of RV pressure load and the methods used to define and characterize RV function and failure. In the future, this may aid in the understanding of the pathobiology of RV failure and development of new therapeutic strategies.

## Abbreviations

CHD: Congenital heart diseases
Ees: End-systolic elastance
Eed: End-diastolic elastance
ERA: Endothelin receptor antagonists
FAO: Fatty acid oxidation
LV: Left ventricle
MCT: Monocrotaline
PAB: Pulmonary artery banding
PDE5: Phosphodiesterase 5
PDK: Pyruvate dehydrogenase kinase
PKG: Protein kinase G
PH: Pulmonary hypertension
RAAS: Renin−angiotensin aldosterone system
RV: Right ventricle
